# Expression Patterns of MicroRNAs in Porcine Endometrium and Their Potential Roles in Embryo Implantation and Placentation

**DOI:** 10.1371/journal.pone.0087867

**Published:** 2014-02-05

**Authors:** Lijie Su, Ruize Liu, Wei Cheng, Mengjin Zhu, Xiaoping Li, Shuhong Zhao, Mei Yu

**Affiliations:** Key Lab of Agricultural Animal Genetics, Breeding and Reproduction of Ministry of Education, Huazhong (Central China) Agricultural University, Wuhan, Hubei, PR China; The University of Georgia, United States of America

## Abstract

Implantation and placentation are critical steps for successful pregnancy. The pig has a non-invasive placenta and the uterine luminal epithelium is intact throughout pregnancy. To better understand the regulation mechanisms in functions of endometrium at three certain gestational stages that are critical for embryo/fetal loss in pigs, we characterized microRNA (miRNA) expression profiles in the endometrium on days 15 (implantation period), 26 (placentation period) and 50 (mid-gestation period) of gestation. The differentially expressed miRNAs across gestational days were detected and of which, 65 miRNAs were grouped into 4 distinct categories according to the similarities in their temporal expression patterns: (1) categories A and B contain majority of miRNAs (51 miRNAs, such as the miR-181 family) that were down- or up-regulated between gestational days 15 and 26, respectively; (2) categories C and D (14 miRNAs) consist miRNAs that were down- or up-regulated between gestational days 26 and 50, respectively. The expression patterns represented by eleven miRNAs were validated by qPCR. The majority of miRNAs were in categories A and B, suggesting that these miRNAs were involved in regulation of embryo implantation and placentation. The pathway analysis revealed that the predicted targets were involved in several pathways, such as focal adhesion, cell proliferation and tissue remolding. Furthermore, we identified that genes well-known to affect embryo implantation in pigs, namely *SPP1*, *ITGB3* and *ESR1*, contain the miR-181a or miR-181c binding sites using the luciferase reporter system. The present study revealed distinctive miRNA expression patterns in the porcine endometrium during the implantation, placentation or mid-gestation periods. Additionally, our results suggested that miR-181a and miR-181c likely play important roles in the regulation of genes and pathways that are known to be involved in embryo implantation and placentation in pigs.

## Introduction

The pig is a litter-bearing species, and the increase in litter size may has a significant impact on the profit potential for swine producers. The prenatal mortality is a major limitation for the increase in the litter size in pigs. Two waves of prenatal mortality occur during the early and mid-gestation in commercial swine, including ∼20–30% of embryonic loss during gestational days 10–30 and ∼10–15% of loss of the remaining fetuses from gestational days 50 to 70 [1]. Recent evidences indicated that the prenatal loss in pigs results mainly from the decreased placental efficiency and uterine capacity [2], [3]. The pig is known to have a non-invasive epitheliochorial placenta which is established as round gestational days 26–30 [4]. In order to maintain sufficient surface area for fetal–maternal exchange, a folded trophoblast/endometrial epithelial bilayer is developed. The trophoblast/endometrial surface area for the maternal-fetal exchange significantly increase from gestational days 35 to 70, coinciding with the rapid growth of the pig fetus [5], [6]. The porcine uterine epithelium is intact throughout pregnancy [7] and the uterine endometrium exhibits characteristic morphological and functional changes, which functions in supporting and nurturing the developing conceptuses during pregnancy. In turn, the hormones, proteases and cellular factors secreted from the conceptuses act directly on the endometrium, and may modify endometrial function to promote the interactions between the uterus and the conceptuses as well as placental development [8]. Thus, gaining an understanding of the molecular mechanisms underlying the uterine endometrium remodeling during pregnancy would be important for the investigating of the molecular basis of the sow prolificacy.

MicroRNAs (miRNAs) are a class of small non-coding RNAs that function as modulators of gene expression by regulating mRNA degradation or by inhibiting translation at the post-transcriptional level [9]. While miRNAs generally inhibit transcript translation, some miRNAs can target specific sites in gene promoters to induce gene expression [10], [11]. Recent reports on miRNA expression profiles suggest that aberrant miRNA expression is associated with human endometrial disorders, such as endometriosis [12]–[14], endometrial hyperplasia and carcinoma [15]–[18]. MiRNAs also can regulate cell-cycle progression during the cyclic changes in the secretory-phase endometrial epithelium [19]. In addition, some differentially expressed miRNAs have been determined in the mouse uterus during peri-implantation period [20], [21]. However, only a few reports have been published on the expression profiles of miRNAs in the porcine placenta or endometrium [22]–[24]. Moreover, the structure of the porcine placenta is different from that of the human and mouse, and therefore, the porcine uterine endometrium likely has different physiological mechanisms in play during pregnancy. The aim of this study is to determine the miRNA expression profiles in porcine endometrium on days 15 (implantation period), 26 (placentation period) and 50 (mid-gestation period) of gestation using the Affymetrix Genechip microRNA array. We found that the majority of miRNAs were expressed at higher levels on gestational day 15. Analysis of the predicted targets revealed that these miRNAs may regulate embryo implantation and placentation. These miRNAs might serve as the biomarkers in the porcine endometrium during pregnancy, and the investigation of their functions in the endometrium will be needed for understanding the prolific natures of sows.

## Results

### Differentially Expressed miRNAs Detected by the Microarray

**Figure 1:**
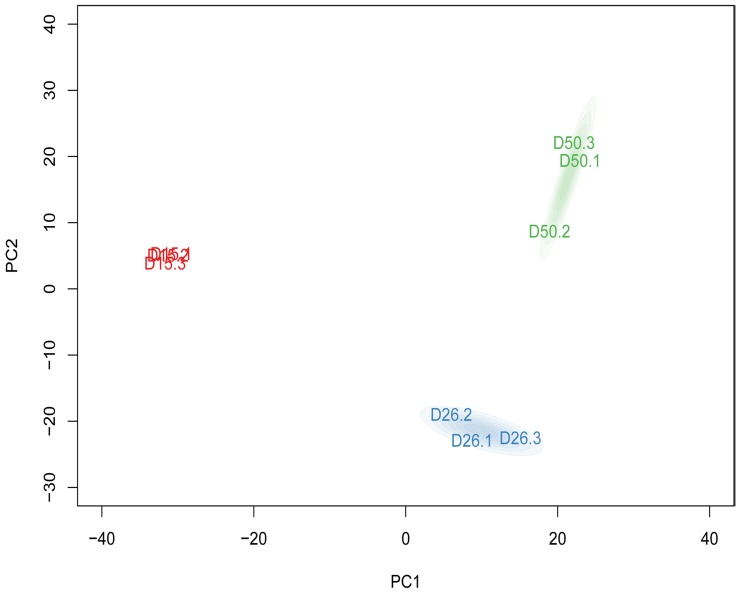
Principal Component Analysis (PCA) plot of microarray data in porcine endometrium. In each case, three replicates from each gestational day cluster together. Clustering of the samples according to gestational day is shown. The red, blue and green circles stand for three different gestational days. D15: gestational day 15; D26: gestational day 26; D50: gestational day 50.

In this study, the Affymetrix miRNA microarray was used to detect miRNA expression profiles in the porcine endometrium during pregnancy. In the initial analysis of the data, we identified similarities and differences between the samples by principal component analysis (PCA). The miRNA expression patterns in samples from the gestational day 15 differed from those in samples from the gestational days 26 and 50 (Figure 1). The 1763 probe sets which represent the human, mouse, rat and porcine mature miRNAs were selected for subsequent analyses (Table S1). After non-specific prefiltering, 513 probe sets (29%) were detected to be expressed in the porcine endometrium (Table S2). The miRNAs showing significant changes in expression (fold change >2, adjusted *p*-value <0.05) in the endometrium between the different days of gestation were summarized in Table 1–3. The number of differentially expressed miRNAs between gestational days 15 and 26 was larger than that between gestational days 26 and 50, suggesting that more miRNAs take place dramatic changes in expression during the implantation and placentation periods.

**Table 1:** The differentially expressed miRNAs in porcine endometrium (gestational day 26 vs gestational day 15).

miRNAs	logFC	adjust-*p*	miRNAs	logFC	adjust-*p*
miR-20a	2.57	1.85E-06	miR-18a	2.81	1.79E-05
miR-126	1.32	4.47E-05	miR-106a	1.66	5.38E-05
miR-17-5p	1.68	6.94E-05	miR-30a-5p	1.28	0.0004
miR-30a-3p	1.33	0.012	miR-15b	1.67	0.018
miR-181d	−3.43	3.13E-07	let-7b	−2.04	8.61E-07
miR-542-5p	−3.02	1.83E-06	miR-135a-star	−4.01	1.85E-06
miR-200c	−1.50	1.85E-06	miR-107	−1.87	1.85E-06
miR-181c	−3.03	2.15E-06	miR-140-3p	−1.94	2.15E-06
let-7c	−1.93	4.31E-06	miR-361	−1.27	1.72E-05
miR-149	−2.07	1.79E-05	miR-31	−1.87	2.42E-05
miR-99b	−1.23	3.45E-05	miR-494	−2.65	4.31E-05
miR-30b-3p	−2.16	4.31E-05	miR-375	−1.47	0.0442
let-7i	−4.41	4.31E-05	miR-125a-3p	−2.84	4.47E-05
miR-320a	−1.57	4.47E-05	miR-181b	−2.68	5.78E-05
miR-214	−2.01	6.25E-05	miR-205	−2.28	8.36E-05
miR-106b-star	−2.82	0.0001	miR-191	−1.09	0.0001
miR-199a-5p	−1.51	0.0001	miR-23b	−1.09	0.0002
miR-145	−1.05	0.0002	miR-139-3p	−2.37	0.0004
miR-874	−2.16	0.0004	miR-210	−2.56	0.0004
miR-92b	−2.72	0.0004	miR-708	−2.54	0.0004
miR-1307	−2.61	0.0005	miR-99b-star	−1.89	0.0009
miR-423-3p	−1.37	0.0011	miR-491-5p	−1.20	0.0013
miR-324-5p	−2.62	0.0013	miR-183	−2.37	0.0022
miR-34b-5p	−1.49	0.0037	miR-339-5p	−1.36	0.0037
miR-181a	−1.11	0.0041	miR-423-5p	−3.25	0.0042
miR-139-5p	−2.83	0.0052	miR-146a	−2.08	0.0071
miR-182	−1.53	0.0093	miR-411	−1.60	0.0174

logFC: log_2_(fold-change).

**Table 2:** The differentially expressed miRNAs in porcine endometrium (gestational day 50 vs gestational day 26).

miRNAs	logFC	adjust-*p*	miRNAs	logFC	adjust-*p*
miR-411	4.10	7.90E-05	miR-487b	4.07	0.0001
miR-215	2.68	0.0043	miR-30b-5p	2.06	0.0048
miR-30c	1.29	0.0131	miR-375	2.06	0.0192
miR-30e	1.36	0.0358	miR-132	−4.05	2.21E-06
miR-221	−3.19	5.63E-05	miR-222	−4.10	8.73E-05
miR-149	−1.67	0.0004	miR-92b	−2.91	0.0011
miR-31	−1.31	0.0016	miR-494	−1.60	0.0071
miR-106a	−1.03	0.0078	miR-34c	−2.72	0.0109
miR-34b-5p	−1.32	0.0192	miR-18a	−1.24	0.0192
miR-106b-star	−1.58	0.0221	miR-92a	−1.33	0.0221
miR-15b	−1.63	0.0386			

logFC: log_2_(fold-change).

**Table 3:** The differentially expressed miRNAs in porcine endometrium (gestational day 50 vs gestational day 15).

miRNAs	logFC	adjust-*p*	miRNAs	logFC	adjust-*p*
miR-30c	2.18	2.14E-05	miR-135a-star	−4.37	8.06E-07
miR-20a	1.70	5.34E-05	miR-106b-star	−4.41	1.14E-06
miR-126	1.53	6.23E-05	miR-92b	−5.63	1.14E-06
miR-30a-5p	1.85	9.89E-05	miR-125a-3p	−3.09	1.14E-06
miR-18a	1.57	0.0002	miR-149	−3.75	1.14E-06
miR-30b-5p	1.83	0.0003	miR-874	−2.91	1.14E-06
miR-30a-3p	1.73	0.0005	let-7i	−4.13	1.34E-06
miR-411	2.50	0.0007	miR-181c	−3.11	1.59E-06
miR-487b	3.22	0.0007	miR-107	−2.53	1.93E-06
miR-215	2.43	0.0019	miR-31	−3.19	2.99E-06
miR-30e	1.22	0.0307	miR-181d	−3.49	2.99E-06
miR-494	−4.26	3.03E-06	miR-708	−3.08	0.0001
miR-132	−3.50	3.03E-06	miR-34c	−4.08	0.0001
miR-140-3p	−2.44	3.03E-06	miR-99b-star	−1.27	0.0001
miR-139-3p	−2.51	5.11E-06	miR-139-5p	−3.75	0.0001
let-7b	−2.04	5.96E-06	miR-222	−4.09	0.0001
miR-542-5p	−3.02	5.96E-06	miR-199a-5p	−1.52	0.0002
miR-221	−3.52	2.96E-05	miR-191	−1.34	0.0003
miR-205	−3.02	3.62E-05	miR-1307	−3.09	0.0004
miR-99b-star	−2.65	3.64E-05	miR-491-5p	−1.45	0.0005
miR-30b-3p	−3.00	4.85E-05	miR-181a	−1.61	0.0006
miR-200c	−1.46	5.28E-05	miR-183	−1.94	0.0008
miR-146a	−1.97	7.02E-05	miR-92a	−1.54	0.0009
miR-210	−2.32	7.02E-05	miR-423-3p	−1.56	0.0009
Let-7c	−1.79	7.02E-05	miR-324-5p	−2.51	0.0009
miR-181b	−3.67	7.29E-05	miR-423-5p	−3.65	0.0020
miR-34b-5p	−2.82	9.06E-05	miR-182	−1.92	0.0020
miR-214	−2.51	9.42E-05	miR-339-5p	−2.20	0.0001
miR-320a	−1.64	0.0001	miR-361-5p	−1.83	0.0001

logFC: log_2_(fold-change).

### Patterns of miRNAs Expression in Porcine Endometrium during Pregnancy

**Figure 2:**
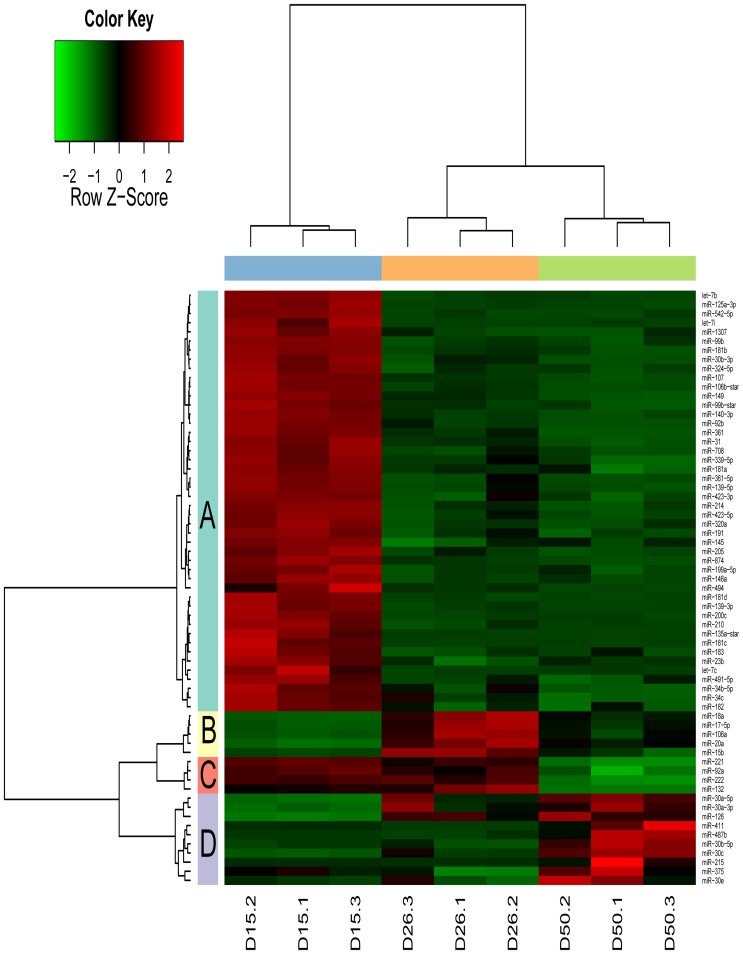
The hierarchical clustering based on differentially expressed miRNAs in porcine endometrium during pregnancy. Each row represents an individual miRNA and each column represents a sample. A color legend indicating the level of miRNA expression is at the top, with red indicating high expression level and green indicating low expression level. D15: gestational day 15; D26: gestational day 26; D50: gestational day 50.

To visually illustrate the expression patterns of miRNAs in the porcine endometrium during pregnancy, we used unsupervised hierarchical clustering to construct a heat map based on the differentially expressed miRNAs. The results showed that 65 miRNAs were grouped into 4 categories (Figure 2). The miRNAs in category A exhibited the high expression levels on day 15 of gestation, and followed by a decrease in expression on gestational days 26 and 50. Category A includes 46 miRNAs, such as miR-205, miR-31, miR-200c, miR-214, miR-320 and miR-494. The miR-181a/b/c/d, belong to the miR-181 family, were also found in category A. The miRNAs in category B were expressed at higher levels on day 26 of gestation compared to those on days 15 and 50 of gestation. Category B contains 5 miRNAs (miR-17, miR-20b, miR-106a, miR-18a and miR-15b). MiR-17, miR-20b, miR-18a and miR-106a are members of the miR-17 family. The miRNAs in category C were expressed at higher levels both on gestational days 15 and 26, and their expression levels decreased on gestational day 50. Four miRNAs (miR-221/222 cluster, miR-132 and miR-92a) are represented for category C. The miRNAs in category D were highly expressed on day 50 of pregnancy. Ten miRNAs, such as miR-215, miR-411, miR-487b and miR-30c, are represented by category D. The expression levels of a large proportion of miRNAs (categories A and B) were differentially expressed between gestational days 15 and 26, suggesting these miRNAs may regulate embryo implantation and placentation in porcine endometrium.

### Analyses of Predicted Targets of the Differentially Expressed miRNAs

**Figure 3:**
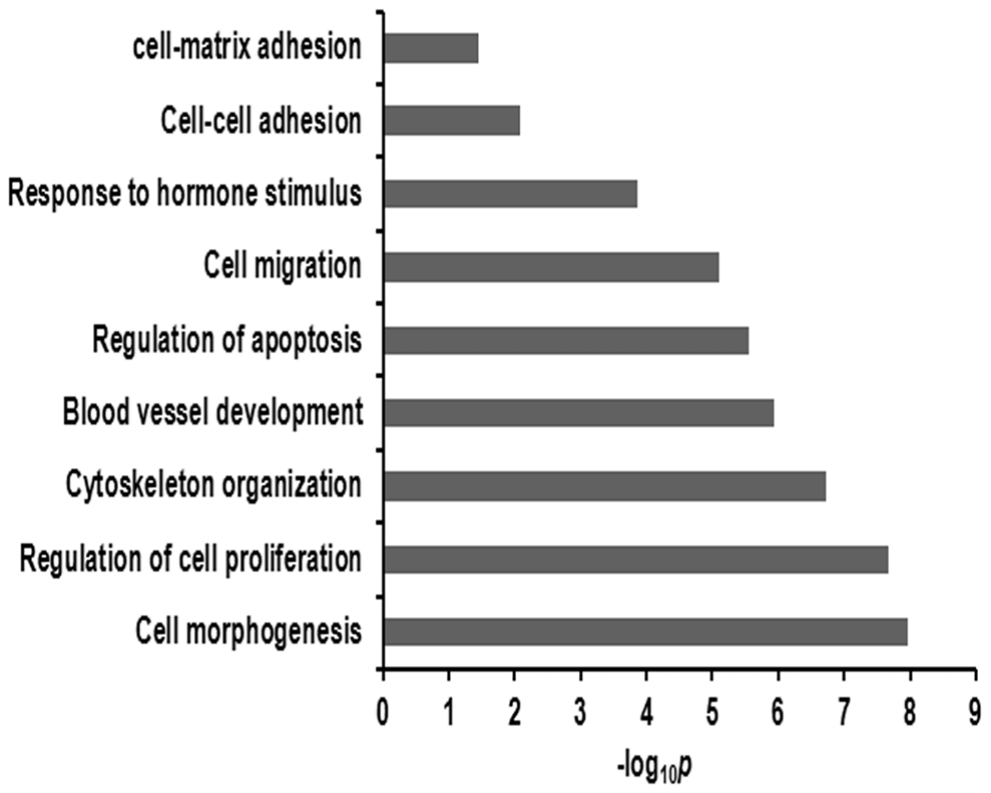
The gene ontology biological process enriched for predicted targets of differentially expressed miRNAs. The negative log of the *p* value (−log_10_ *P*) was plotted on the x-axis.

**Figure 4:**
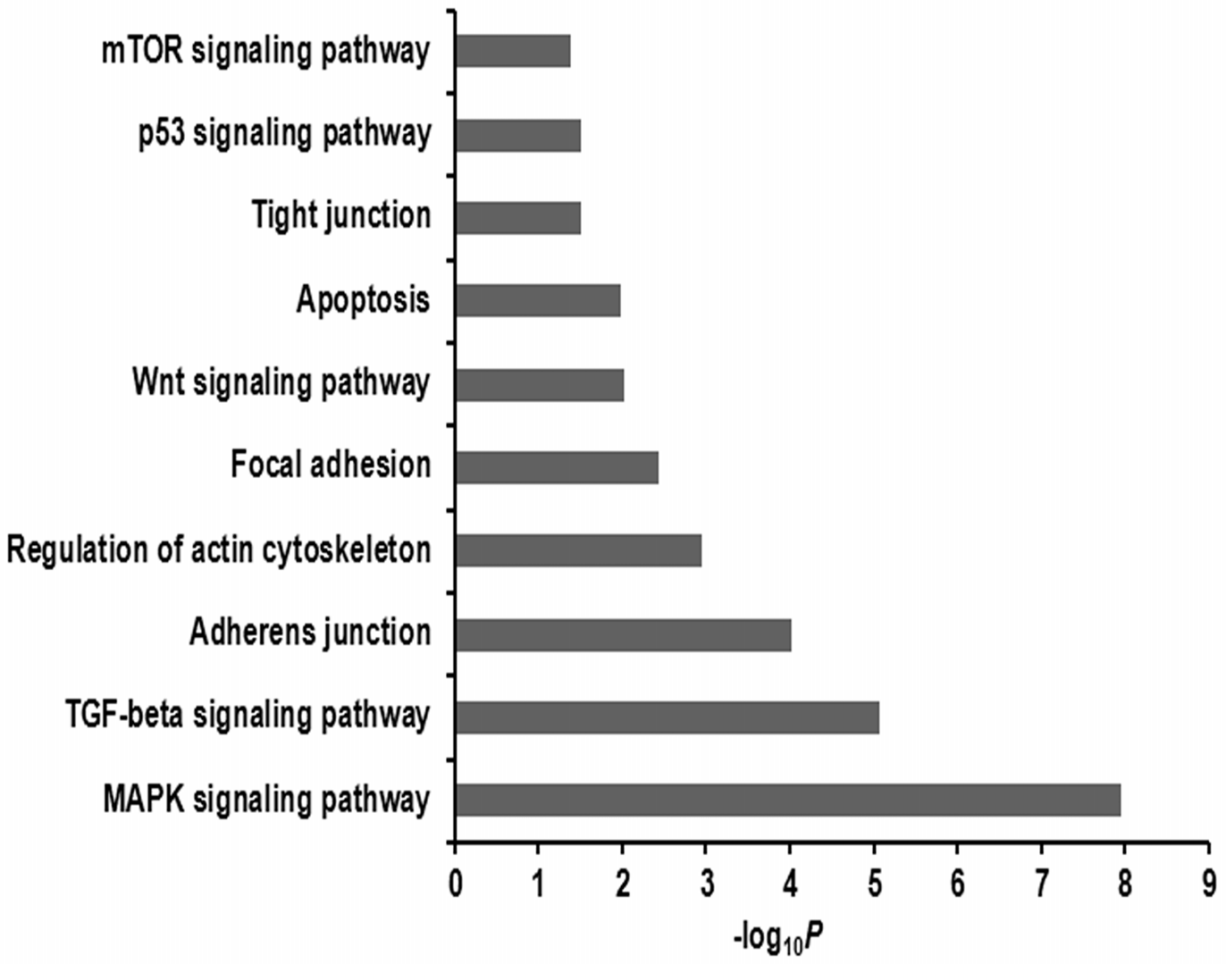
The KEGG pathways enriched for predicted targets of differentially expressed miRNAs. The negative log of the *p* value (−log_10_ *P*) was plotted on the x-axis.

To gain insight into the possible regulatory role of miRNAs, we predicted the targets for the differentially expressed miRNAs using three softwares (TargetScan, miRanda and miRDB). Only targets identified by all the three softwares were considered to be the predicted targets for each miRNA. An *in silico* analysis identified 3745 predicted targets for the differentially expressed miRNAs (Table S3). Some predicted targets were the well-studied genes known to regulate embryo implantation and placentation in pigs, such as miR-181a/c-*SPP1*, miR-181a/c-*ITGB3*, miR-181c-*ESR1*, miR-17-*STC1* and miR-31-*FGF7*. Gene ontology biological process analysis based on the predicted targets showed that the differentially expressed miRNAs were involved in remodeling of the endometrium (e.g., “regulation of cell proliferation”, “cell migration” “regulation of apoptosis”, “cytoskeleton organization”, “blood vessel development”) and different aspects of cell communication (e.g., “response to hormone stimulus”, “cell-cell adhesion” and “cell-matrix adhesion”) (Figure 3 and Table S4). The KEGG pathway analysis based on predicted targets revealed that differentially expressed miRNAs were involved in several pathways, such as focal adhesion, mitogen-activated protein kinase (MAPK) signaling pathway, TGF-beta signaling pathway, Wnt signaling pathway, regulation of actin cytoskeleton, adherens junction, tight junction and mTOR signaling pathway (Figure 4 and Table S5).

### Validation of the miRNA Candidates by RT-qPCR

**Figure 5:**
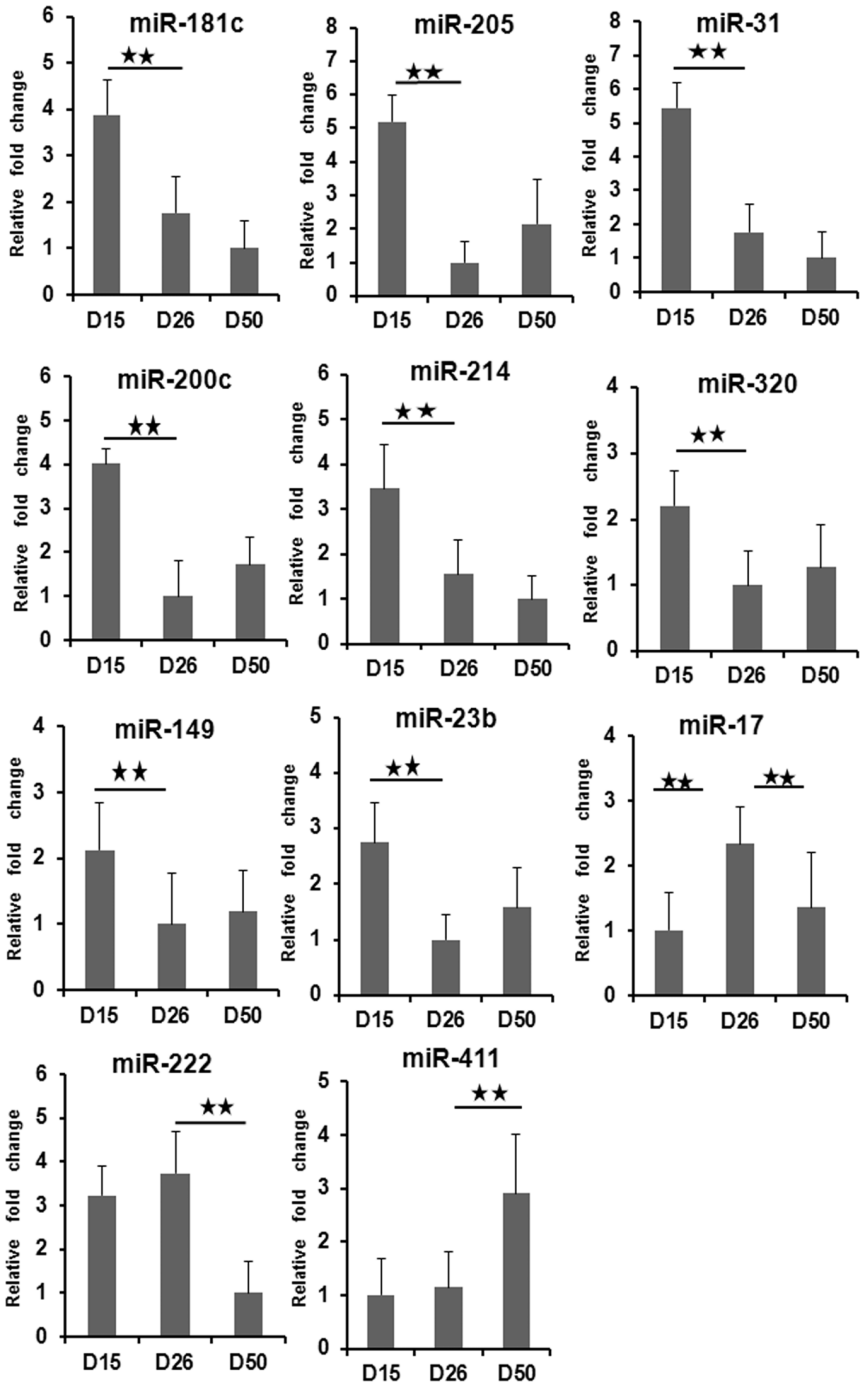
Validation of differentially expressed miRNAs by qPCR. All samples were normalized to RNU6. The error bars show the SEM. The significance of differences for miRNA expression was calculated using two-tailed T-test. *, *p*<0.05; **, *p*<0.01. D15: gestational day 15; D26: gestational day 26; D50: gestational day 50.

Eleven miRNAs (miR-205, miR-222, miR-200c, miR-23b, miR-411, miR-17, miR-31, miR-149, miR-320, miR-181c and miR-214) were chosen for validation by RT-qPCR. The results are shown in Figure 5. In general, the expression patterns for the eleven miRNAs were consistent with those in the microarray data. MiR-205, miR-214, miR-200c, miR-181c, miR-149, miR-23b, miR-320 and miR-31, which belong to category A, showed the higher expression levels on day 15 of gestation. MiR-17 in category B were higher expression levels on day 26 of pregnancy. MiR-222 in category C showed the higher expression level both on gestational days 15 and 26. The expression levels of miR-411 in category D increased in endometrium on day 50 of gestation.

### Validation of the Binding Sites of miR-181a and miR-181c Exist in 3′UTR of Three Genes

**Figure 6:**
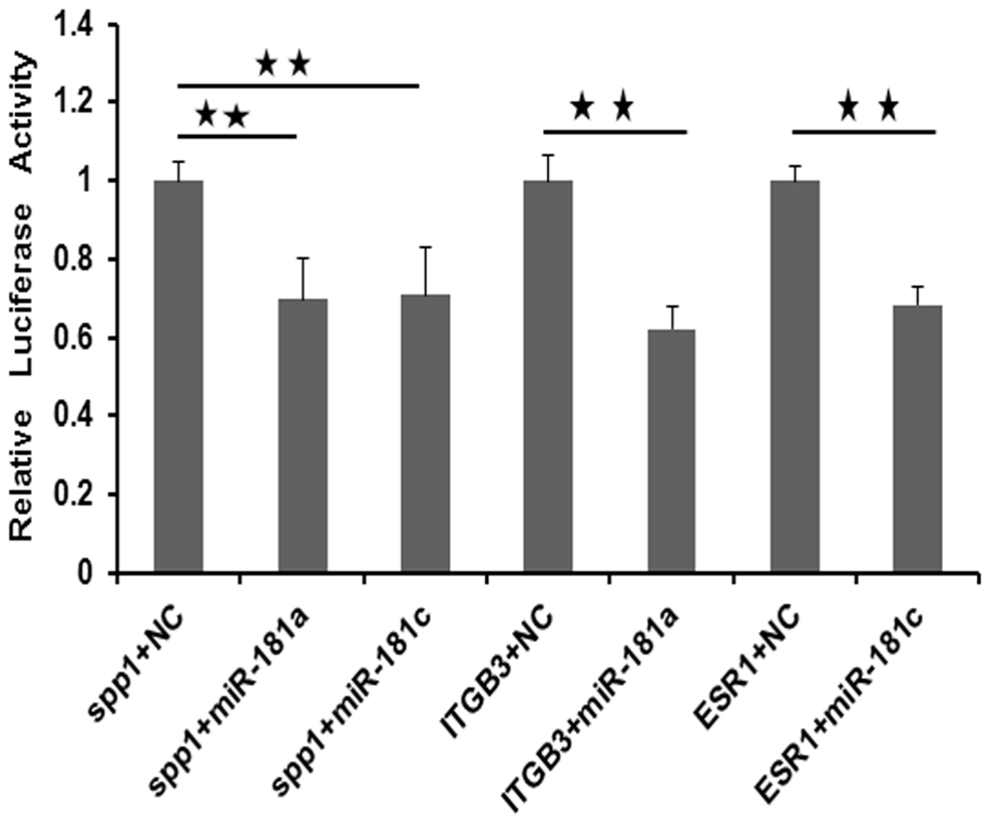
The result of luciferase assay for *SPP1*, *ITGB3* and *ESR1* targeting by miR-181a and miR-181c. The error bars show the SEM. The significance of differences was calculated using two-tailed T-test. *, *p*<0.05; **, *p*<0.01.

MiR-181a and miR-181c were down-regulated between gestational days 15 and 26, suggesting that they may be involved in regulation of embryo implantation. Three mostly well-studied genes associated with embryo implantation in pigs, namely *SPP1*, *ITGB3* and *ESR1*, were the predicted targets of miR-181a and miR-181c by using three available target prediction tools. Secreted phosphoprotein 1 (SPP1), a secretory glycoprotein, known to mediate conceptus trophoblast attachment, growth, and epitheliochorial placentation in pigs [25]–[27]. Integrin beta 3 (ITGB3), an adhesion molecule, mediates cell adhesion in implantation process [28]. Estrogen receptor 1 (ESR1), a ligand-activated transcription factor, is the primary driver of estrogen action that required for embryo implantation in pigs [29]. To validate that *SPP1*, *ITGB3* and *ESR1* were putative targets of miR-181a or miR-181c in the pig, the 3′UTR of porcine *SPP1*, *ITGB3* and *ESR1* containing the miR-181 binding sequences were cloned into the psi-CHECK™-2 dual luciferase reporter plasmid, respectively. The reported plasmids along with miR-181a or miR-181c mimic were co-transfected into PK-15 cells. A scrambled sequence was constructed for negative control. The luciferase activity was significantly attenuated in cells transfecting with miR-181a or miR-181c mimic compared to the negative control (Figure 6). These results indicated that *SPP1*, *ITGB3* and *ESR1* were putative targets of miR-181a and miR-181c in the pig.

## Discussion

The present study investigated miRNA expression profiles in the porcine endometrium during the implantation, placentation and mid-gestation periods using microRNA microarray. Four categories of expression patterns were distinguished and we noted that a large proportion of miRNAs showed altered expression levels (category A and B) during the implantation and placentation periods. In addition, Gene Ontology biological process analysis based on predicted targets showed that these miRNAs were involved in various processes, including cell proliferation, cell migration, cell adhesion, and cytoskeleton organization. Implantation is the process that the attachment of the developing blastocyst to the uterus wall for establishing a functional placenta and successful pregnancy [30]. In pigs, most embryonic loss occurs during the implantation and placentation periods. A critical event of embryo implantation and placentation is the extensive tissue remodeling at the maternal-fetal interface, which is characterized by cell proliferation, cell migration, and cell adhesion [31]. Therefore, our results revealed that miRNAs may play an important role in regulating of porcine endometrial remodeling in response to embryo implantation and placentation.

Furthermore, we found some miRNAs targeted the well-studied genes which are critical for embryo implantation in pigs, such as miR-181a/c-*SPP1*, miR-181a/c-*ITGB3*, miR-181c-*ESR1*, miR-17-*STC1*, and miR-31-*FGF7*. Secreted phosphoprotein 1 (SPP1), a highly phosphorylated acidic glycoprotein, has been demonstrated to participate in attachment of conceptus trophoblast to uterine luminal epithelium, and mediates epitheliochorial placentation in pigs [25]–[27]. The miR-181a has been demonstrated to be a target of *SPP1* gene to regulate metastatic function in hepatocellular cancer cell lines [32]. Previous reports revealed that the SPP1 protein is detectable on the uterine luminal epithelium on day 12 of pregnancy and the level is increased on day 30 in pigs [25]. Coincidentally our data showed that the expression levels of miR-181a and miR-181c were down-regulated between gestational days 15 and 26. Thus, the SPP1 protein level is correlated inversely with the expression levels of miR-181a and miR-181c in porcine endometrium. In addition, we identified that miR-181a and miR-181c can specifically bind to the 3′UTR of *SPP1* gene by luciferase reporter system. Therefore, these results suggested that the miR-181a and miR-181c may target *SPP1* gene in porcine endometrium to regulate embryo implantation and epitheliochorial placentation. Integrin beta 3 (ITGB3) is an adhesion molecule and is expressed in a punctuate pattern only on the apical surface of uterine luminal epithelium during early pregnancy in pigs [27], [28]. It has been shown that ITGB3 and ITGAV subunits together bind to SPP1 to generate stable adhesions between the maternal-fetal interface in pigs after the initial attachment of conceptus trophoblast to the endometrial luminal epithelium [26], [27]. We also found that *ITGB3* was putative target of miR-181a by luciferase reporter system, suggesting that the miR-181a could be the regulator for the stable adhesion between the maternal-fetal interface during pregnancy. The endometrium is a complex steroid-responsive tissue that undergoes cell proliferation and differentiation in preparation for embryo implantation [33]. In pigs, conceptuses secrete estrogen to provide the initial maternal recognition signal and can mediate regulation in endometrial remodeling through the presence of estrogen receptor ESR1 [29], [34]. We identified that *ESR1* gene contains the miR-181c binding site by luciferase reporter system, suggesting a role of miR-181c in regulation of estrogen-mediated gene expression and influencing implantation in pigs. Stanniocalcin 1 (STC1), a luminal epithelial marker during embryo implantation in pigs, plays a role in regulating uterine receptivity for conceptus attachment in implantation process [35]. *STC1* mRNA is abundant in endometrium luminal epithelium on days 12–20 of pregnancy and the expression level is decreased on day 25 of pregnancy [35]. In contrast, our results of microarray and qPCR investigation showed that the expression level of miR-17 was up-regulated between gestational days 15 and 26. Furthermore, *STC1* was a predicted target of miR-17, implying that miR-17 could be a potential regulator for uterine receptivity in pigs. Fibroblast growth factor 7 (FGF7), a growth factor secreted by uterine luminal epithelium, plays a role in stimulating the proliferation, differentiation and migration of conceptus trophectoderm during the implantation period in pigs [36]. MiRNA target algorithms predicted that *FGF7* was a target of miR-31. Thus, miR-31 may influence conceptus survival and growth during the implantation period by targeting *FGF7*. Taken together, our findings revealed miRNAs that may regulate genes which have well-known functions in embryo implantation and placentation in pigs.

*SPP1* and *ITGB3*, which were the putative targets of miR-181a and miR-181c, are components of focal adhesion signal pathway. Focal adhesions are dynamic macromolecular complexes comprised of integrins which link the extracellular matrix (ECM) to the actin cytoskeleton [37] and have been demonstrated to play an important role in implantation process. The implantation process is classified into three phases: apposition, attachment and invasion [38]. The dynamics of focal adhesions influence the process of cell attachment on uterine luminal epithelium in rat and ovine uterus, as well as embryo invasion in humans [39]–[41]. Many of components of focal adhesion signal pathway link integrin-mediated signals with other signaling pathways, such as mTOR, PI3K, MAPK signaling pathway [42]. The miR-181a and miR-181c may regulate embryo implantation and placentation by regulating the focal adhesion signaling pathway. Moreover, we found that many miRNAs included in the category A were predicted to target the components of focal adhesion signal pathway. For example, Talin, that is encoded by the *TLN1* gene, is a key component of focal adhesions and an important regulator of integrin activation [43]. *TLN1* was predicted to be a target of miR-200c. MiR-107 may target *VCL*, which encodes a cytoskeletal protein of focal adhesion, to regulate the linkage of integrins to the actin [44]. Focal adhesion kinase (FAK), a protein tyrosine kinase, is recruited to focal adhesions and mediates many of the downstream responses [45]. And previous report demonstrated that miR-205 can inhibit the expression of FAK in renal cancer [46]. In addition, cytoskeletal reorganization is essential for the attachment of the conceptus trophectoderm to the endometrial luminal epithelium [47]. Rho GTPases, including RhoA, Rac1 and CDC42, which can control the cytoskeletal changes by linking ECM molecules to the actin cytoskeleton for focal adhesion assembly [48]. *RhoA* is a validated target of miR-31 [49]. MiR-31 was also predicted to target *CDC42*. We speculated that miR-31 may be involved in cytoskeletal reorganization in porcine endometrium, which is critical for remodeling of endometrium during the implantation period. In summary, the multiple miRNAs which in category A may influence embryo implantation and placentation by regulation of the focal adhesion signal pathway.

miRNAs in categories B, C and D were differentially expressed between gestational days 26 and 50. Coinciding with the two critical periods for placentation and placental development, the trophoblast/endometrial surface area has been observed to increase markedly to maintain sufficient surface area for fetal–maternal exchange [2], [50]. Many miRNAs in categories B, C and D have been identified to regulate the expression of genes function in cell proliferation and angiogenesis. The miR-17 was included in category B. The E2F transcription factor (E2F1), which plays a central role in cell-cycle progression, was found to be regulated by miR-17 [51]. The miR-221 and miR-222 in categoriy C have been determined to have the ability to regulate the cell proliferation by inhibition of the cell cycle repressor cyclin-depenent kinase inhibitor 1 B (p27) [52]. The miR-126, included in category D, is a key positive regulator of angiogenic signaling in endothelial cells. MiR-126 enhances the vascular endothelial growth factor (VEGF) and fibroblast growth factor (FGF) signalling via repression of inhibitors of these pathways, leading to angiogenesis and vasculature development [53], [54]. VEGF may contribute to the increase in the growth of the placental vasculature as gestation advances to meet the requirement of the rapidly growing pig fetus [55]. Therefore, miRNAs in categories B, C and D may regulate placentation and placental development by targeting genes associating with cell proliferation and angiogenesis.

### Conclusion

The present study revealed the distinctive miRNA expression patterns in the porcine endometrium on days 15, 26 and 50 of gestation and miRNAs that might play an important role in the embryo implantation and placentation. The results provided a better understanding of the role of miRNA in the porcine endometrium remodeling during the three stages of gestation.

## Materials and Methods

### Ethics Statement

All research involving animals were conducted according to the regulation (No. 5 proclaim of the Standing Committee of Hubei People’ Congress) approved by the Standing Committee of Hubei People’ Congress, P. R. China. Sample collection was approved by the ethics committee of Huazhong Agricultural University. Animals were humanely sacrificed as necessary to ameliorate suffering. Electrocution were used for humane form of euthanasia.

### Sample Collection and RNA Preparation

Yorkshire gilts were obtained from the pig farm of Huazhong Agricultural University (Wuhan, China). The gilts in estrus were bred naturally twice, 24h apart and were euthanized at the following days: gestational day 15, gestational day 26 and gestational day 50, considering that gestational days 15, 26 and 50 are three important times points for the implantation, placentation and mid-gestation periods. The first day of mating was considered to be Day 0 of pregnancy. The pregnancy was confirmed by the presence of normal conceptus in the uterine flushing (gestational day 15) or upon hysterectomy (gestational days 26 and 50). Each uterus was opened longitudinally at the anti-mesometrial site. For pigs on gestational day 15, three samples of the endometrium were randomly collected from the anti-mesometrial sites. For pigs on gestational days 26 and 50, the endometrium samples at three healthy conceptus-attachment sites were collected. All tissue samples were washed briefly with PBS and then immediately frozen in liquid nitrogen. Total RNA from the endometrium samples were extracted using Trizol reagent (Invitrogen, Carlsbad, California, United States). The quality and quantity were determined by electrophoresis and spectrophotometry, and then were sent for hybridization to the miRNA Affymetrix microarray in a commercial service. For microarray hybridization, each RNA sample from one endometrial site of one gilt was pooled in equal volume with that from the corresponding site of the other gilt on each gestational day. Therefore, three pools of RNA samples from three different conceptus-attachment sites of the two gilts on each gestational day were used for the microarray hybridization. The RT-qPCR was performed on three endometrial samples from each gilts (n = 3–4 gilts/gestational day) to validate the results of microarray data.

### Affymetrix GeneChip® miRNA Arrays

The affymetrix GeneChip® miRNA 1.0 Array (Affymetrix, Santa Clara, California, United States) were processed using a commercial Affymetrix array service (GeneTech Biotechnology Limited Company, Shanghai, China). The affymetrix GeneChip® miRNA 1.0 Array contains 46,228 probes, comprising 7,815 probe sets, and covers 71 organisms including human, mouse, rat and pig. The content is derived from the Sanger miRBase miRNA database v11.0 (http://www.mirbase.org). The RNA samples were labeled with the Genisphere FlashTag Biotin HSR RNA labeling kit (Genisphere, Hatfield, United Kingdom). The labeled RNA was quantified, fractionated and hybridized to the miRNA microarray according to the standard procedures provided by the manufacture. The chips were washed and stained using a Genechip Fluidics Station 450 (Affymetrix, Santa Clara, California, United States). The chips were then scanned with an Affymetrix GeneChip Scanner 3000 (Affymetrix, Santa Clara, California, United States).

### Analysis of the Affymetrix miRNA Arrays Data

Data from the Affymetrix CEL files were loaded into R program using the Affy package. After subtraction of background intensities and normalization by RMA method [56], the Log2 transformed intensities were obtained. The microarray data had been deposited in NCBI’s Gene Expression Omnibus (GEO) and the accession number is GSE35995 (http://www.ncbi.nlm.nih.gov/geo/query/acc.cgi?token=jpkbhuciyygukpc&acc=GSE35995). The principal component analysis (PCA) plot of samples was performed using all probe sets, by using a median centering of the data set. The 1763 probe sets which represent the human, mouse, rat and porcine mature miRNAs were selected for subsequent analyses. To ensure specificity of miRNAs detection in porcine endometrium, only the 842 probe sets homologous with the porcine miRNAs in miRbase were used for non-specific prefiltering on the basis of variability [57]. The probe sets with the maximum of normalization value <4 or with standard deviation <0.2 across samples were filtered out. Therefore, the filtered data sets consisted of 513 probe sets which represent 261 miRNAs. All the filtered probe sets were used for identification of the differentially expressed miRNAs by the linear models and the empirical Bayes methods (Limma R package). The raw *P* values were adjusted by the Benjamini-Hochberg false discovery rate to yield adjusted *P* values. The criteria for identification of the differentially expressed miRNAs were established as a fold change >2 with an adjusted *P*<0.05. Unsupervised hierarchical clustering analyses for differentially expressed miRNAs were performed by using the R language. Correlation similarity matrix and complete linkage algorithms were used in the cluster analysis.

### Bioinformatics Analysis

Target genes of the differentially expressed miRNAs were predicted by using three available target prediction programs, namely miRanda (http://www.microrna.org/microrna/home.do), TargetScan (http://targetscan.org/), miRDB (http://mirdb.org/miRDB/). Only target genes identified by all the three databases were considered to be predicted target genes for each differentially expressed miRNA. The Gene Ontology biological process and KEGG pathway analyses for the predicted targets were performed using the DAVID (Database for Annotation, Visualization and Integrated Discovery) web-based tool (http://david.abcc.ncifcrf.gov/).

### Real-time Quantitative PCR of Mature miRNAs

RT-qPCR was used to validate the results of microarray data. Primers (Table 4) were designed on the basis of miRNA mature sequence. Total RNA was reversely transcribed using One Step PrimeScript miRNA cDNA Synthesis Kit (TakaRa, Dalian, China) according to the manufacturer’s instructions. The ploy (A) was added to the 3′ end of miRNAs. A primer consisting of an oligo(dT) sequence is used for reverse transcription. QPCR was performed using SYBR Premix Ex Tag II (Takara, Dalian,China) in the LightCycler 480 Real-Time PCR machine (Roche, Basel, Switzerland). PCR conditions were as follows: single cycle of 5 min at 95°C, followed by 40 cycles of 30sec at 95°C, 20 sec at 60°C, and 15 sec at 72°C. Small nuclear RNA U6 was used as internal control. All qPCRs were performed in triplicate. The 2^−ΔΔ*C*T^ (“delta-delta Ct”) method was used to determine the differences in expression between the different comparisons. The differences in miRNA expression levels between groups were compared using the two tailed T-test. A *p* value <0.05 was considered significant.

**Table 4:** Primers used for qPCR validation in porcine endometrium.

miRNA	Sequence	Tm(°C)
miR-205	TCCTTCATTCCACCGGAGTCTG	60
miR-222	AGCTACATCTGGCTACTGGGTCTC	60
miR-200c	TAATACTGCCGGGTAATGATGGA	60
miR-23b	ATCACATTGCCAGGGATTACCA	60
miR-411	TAGTAGACCGTATAGCGTACG	60
miR-17	CAAAGTGCTTACAGTGCAGGTAG	60
miR-31	AGGCAAGATGCTGGCATAGCTG	60
mir-149	TCTGGCTCCGTGTCTTCACTCCC	60
miR-320	AAAAGCTGGGTTGAGAGGGCGAA	60
miR-181c	AACATTCAACCTGTCGGTGAGT	60
miR-214	TACAGCAGGCACAGACAGGCAG	60

The universal reverse primer and the primer of RNU6 gene were provided from One Step PrimeScript miRNA cDNA Synthesis Kit (TaKaRa, Dalian, China).

### Dual Luciferase Reporter Assays

The 3′UTR of *SPP1*, *ITGB3* and *ESR1* containing the miR-181a and miR-181c binding sequence was cloned into the psiCHECK™-2 dual luciferase reporter plasmid (Promega, Fitchburg, Wisconsin, United States). The PCR primer for *SPP1* plasmid were forward (5′-CCGCTCGAGTGAGAATTGCAGTGATAGC-3′) and reverse (5′-AATGCGGCCGCCCTCTCTTATAAGTGTGTC-3′); The PCR primer for *ITGB3* plasmid were forward (5′-CCGCTCGAGCCAGAGCCAAATGGGACACA-3′) and reverse (5′-AATGCGGCCGCGATCAGAGAGCCCTTACAGACC -3′);The PCR primer for *ESR1* plasmid were forward (5′-CTCGAGCTAAGGCTTCTCTTGGGAT-3′) and reverse (5′-GCGGCCGCCTGGTATTACATCATCTAT-3′). The accuracy of the plasmid inserts was determined by complete sequencing analysis. The miRNA mimics were synthesized as duplexes. The miRNA mimic sequences: ssc-miR-181a (5′-AACAUUCAACCUGUCGGUGAGU-3′); ssc-miR-181c (5′-AACAUUCAACGCUGUCGGUGAGUU-3′). A scrambled sequence (NC) was constructed for negative control. The NC sequence: 5′-UUCUCCGAACGUGUCACGUTT-3′. PK15 cells were culture in DMEM complete medium (Hyclone, Logan, Utah, United States) supplemented with 10% FBS (Hyclone, Logan, Utah, United States) and 1% penicillin/streptomycin (Hyclone, Logan, Utah, United States). For the reporter analysis, PK-15 cells were seeded in 24-well plates 24 hours prior to transfection. At the following day, 200ng of reporter plasmid along with 50nM of miR-181a or miR-181c mimic were co-transfected using Lipofectamine 2000 (Invitrogen, Carlsbad, California, United States). Cells were collected 24 hours after transfection and luciferase activity was measured using the dual luciferase reporter assay system (Promega, Fitchburg, Wisconsin, United States), and the Renilla luciferase activity was normalized to the firefly luciferase activity.

## Supporting Information

Table S1 **A list of the 1763 probe sets which represent the human, mouse, rat and porcine miRNAs.**
(XLSX)

Table S2 **A list of the 513 probe sets after non-specific filtering.**
(XLSX)

Table S3 **A list of predicted targets of the differentially expressed miRNAs.**
(XLSX)

Table S4 **A list of Gene Ontology enriched for predicted targets of the differentially expressed miRNAs.**
(XLSX)

Table S5 **A list of KEGG pathways enriched for predicted targets of the differentially expressed miRNAs.**
(XLSX)
